# Composite hemangioendothelioma- report of two cases located in bone and review of the literature

**DOI:** 10.1186/s12891-023-06745-8

**Published:** 2023-08-24

**Authors:** Yunyang Deng, Mei Li

**Affiliations:** Department of Radiology, Shanghai Sixth People’s Hospital Affiliated to Shanghai Jiao Tong University School of Medicine, Shanghai, China

**Keywords:** Composite hemangioendothelioma, Hemangioendothelioma, Imaging diagnosis, PET-CT, Pelvis, Vertebrae

## Abstract

**Background:**

Composite hemangioendothelioma (CHE) is a rare intermediate-grade vascular tumor characterized by a complex histologic component. It occasionally metastasizes, but local recurrence is not uncommon. CHE is mainly located in the extremities’ distal dermis and subcutaneous soft tissues. It is rarely located in the bone. We report here two cases originally occurred in bone.

**Case presentation:**

The first case of CHE occurred solely on the left pubis. The second case is a patient post-resection of CHE in the manubrium sterni 10 years ago [1], who presented with multiple lesions in the left ilium and T6, T12 vertebra. All these lesions in the two cases showed osteolytic bone destruction on computed tomography (CT) scans and showed relatively high signal intensity on the fat-suppressed sequences of T2-weighted magnetic resonance (MR) images and isointense signal intensity on T1-weighted MR images. After injection of contrast agent (Gd-DTPA), the lesions showed inhomogeneous enhancement. 2-deoxy-2 [F-18] fluoro-D-glucose positron emission tomography-computed tomography (^18^FDG PET-CT) showed increased FDG uptake in these osteolytic bone destruction areas with SUVmax around 5.0. Both patients underwent surgery. Lesions in the left pubis and left ilium were confirmed by postoperative pathology while lesions on the vertebrae were only biopsied, not surgically resected. The first patient had no recurrence or metastasis in 5 years after surgery. The second patient had surgery recently and is still being followed up.

**Conclusions:**

CHE occurring in bone is rarely reported. Our report provides more detailed information on the diagnosis of CHE. Given that CHE is locally aggressive and occasionally metastatic, PET-CT may be helpful in staging and follow-up.

## Background

Composite hemangioendothelioma (CHE) is a recently described vascular tumor [2]. CHE is classified as an intermediate-grade vascular tumor in the 5th edition of the World Health Organization (WHO)classification of soft tissue and bone tumors released in 2020 [3]. It is defined as a locally aggressive, occasionally metastatic vascular tumor containing a mixture of histologically distinct components [4]. CHE is very rare, with about 59 cases reported in the English literature so far [5]. Herein, we report two cases of CHE, the first case occurred in the left pubis (female, 21 years old), and the second case is a patient post-resection of CHE in the manubrium sterni 10 years ago [1], who developed of new lesions in the left ilium and T6, T12 vertebra (male, 66 years old). To the best of our knowledge, it is the 3rd report describing CHE occurring originally in bone [1, 6]. These cases highlighted the relative frequency of occurrence in bone. Due to the specialty and complexity of its pathology, most of the previously reported cases focused on the description of the pathology and Immunohistochemistry, while descriptions of the imaging manifestations of CHE were rarely reported. This article is focused on the analysis of imaging presentation and aimed to provide more detailed information for the diagnosis of CHE.

## Case presentation

### Case 1

**Fig. 1:**
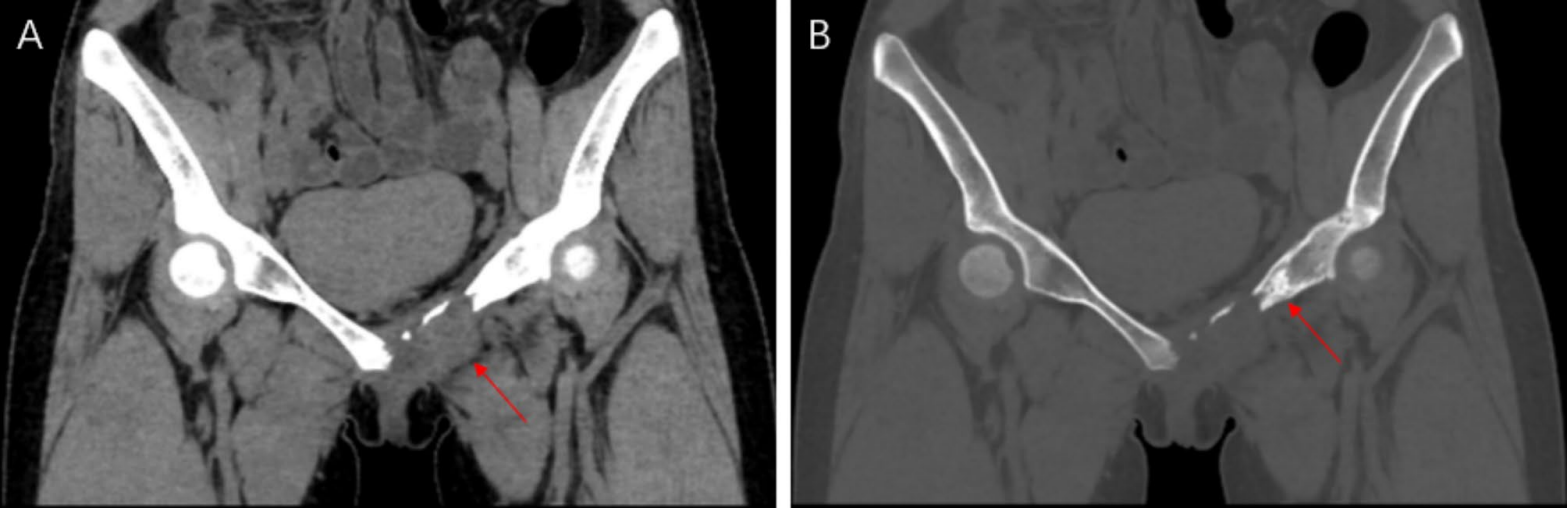
CT revealed a lesion of osteolytic bone destruction in the left pubis with a soft tissue mass (**A**, arrow), surrounded by local reactive osteosclerosis (**B**, arrow). And the bone cortex surrounding the lesion was damaged

**Fig. 2:**
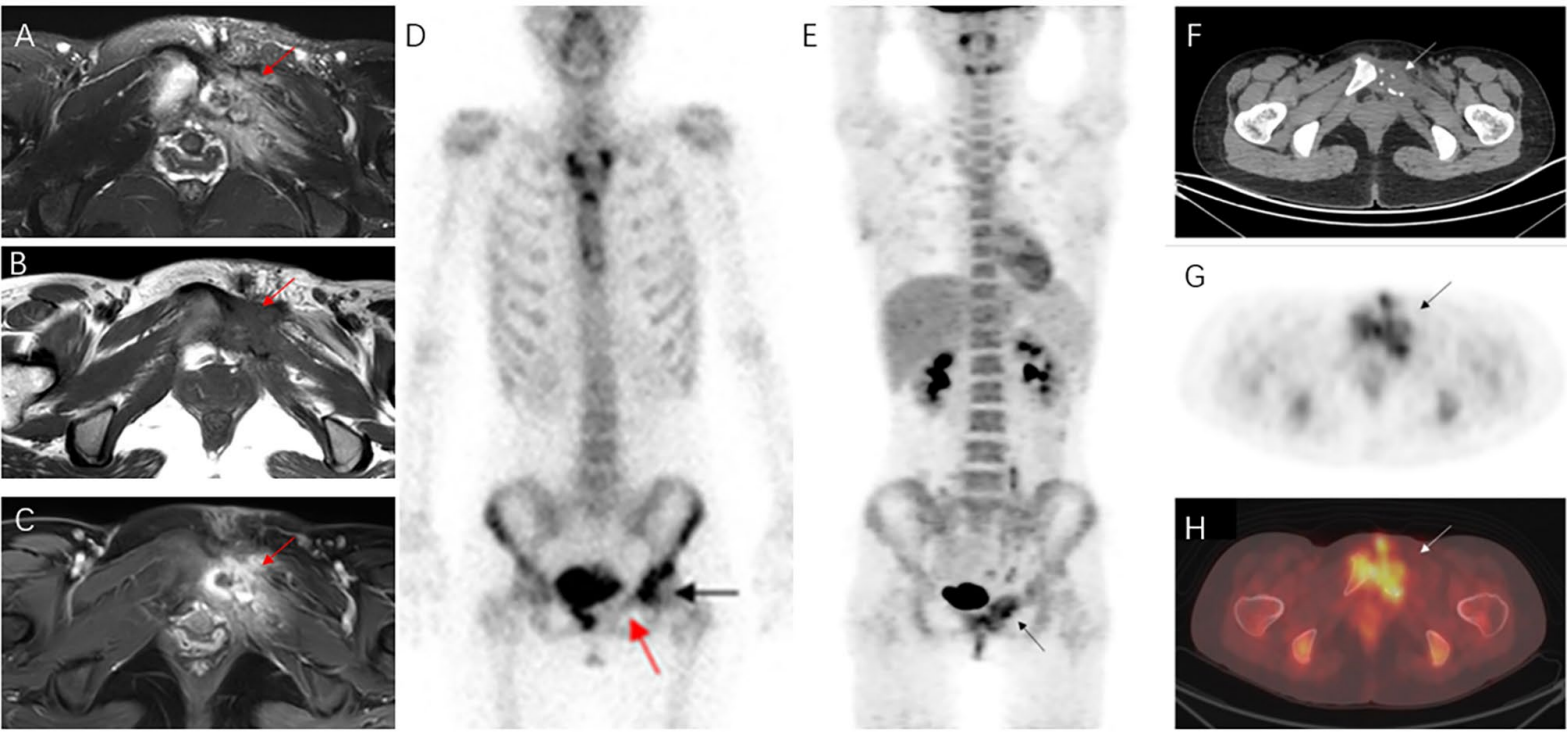
The lesion showed high signal intensity on the fat-suppressed sequences of T2-weighted MR images (**A**, arrow) and isointense signal intensity on T1-weighted MR images (**B**, arrow), with a size of about 36 mm×35 mm. After injection of the contrast agent (Gd-DTPA), the lesion showed inhomogeneous enhancement (**C**, arrow). Bone marrow edema of the right pubis was shown on MRI. A 99mTc MDP bone scan showed that this lesion was radioactively absent (**D**, red arrow), while the adjacent left acetabulum was radioactively concentrated because of reactive osteosclerosis (D, black arrow). The maximum intensity projection (MIP) PET (**E**), transverse CT (**F**), corresponding PET (**G**), and fusion (**H**) images showed increased FDG uptake of the osteolytic lesion (arrows) with SUVmax of 5.0. Except for this lesion, no abnormal FDG uptake was observed in any other parts of the body

**Fig. 3:**
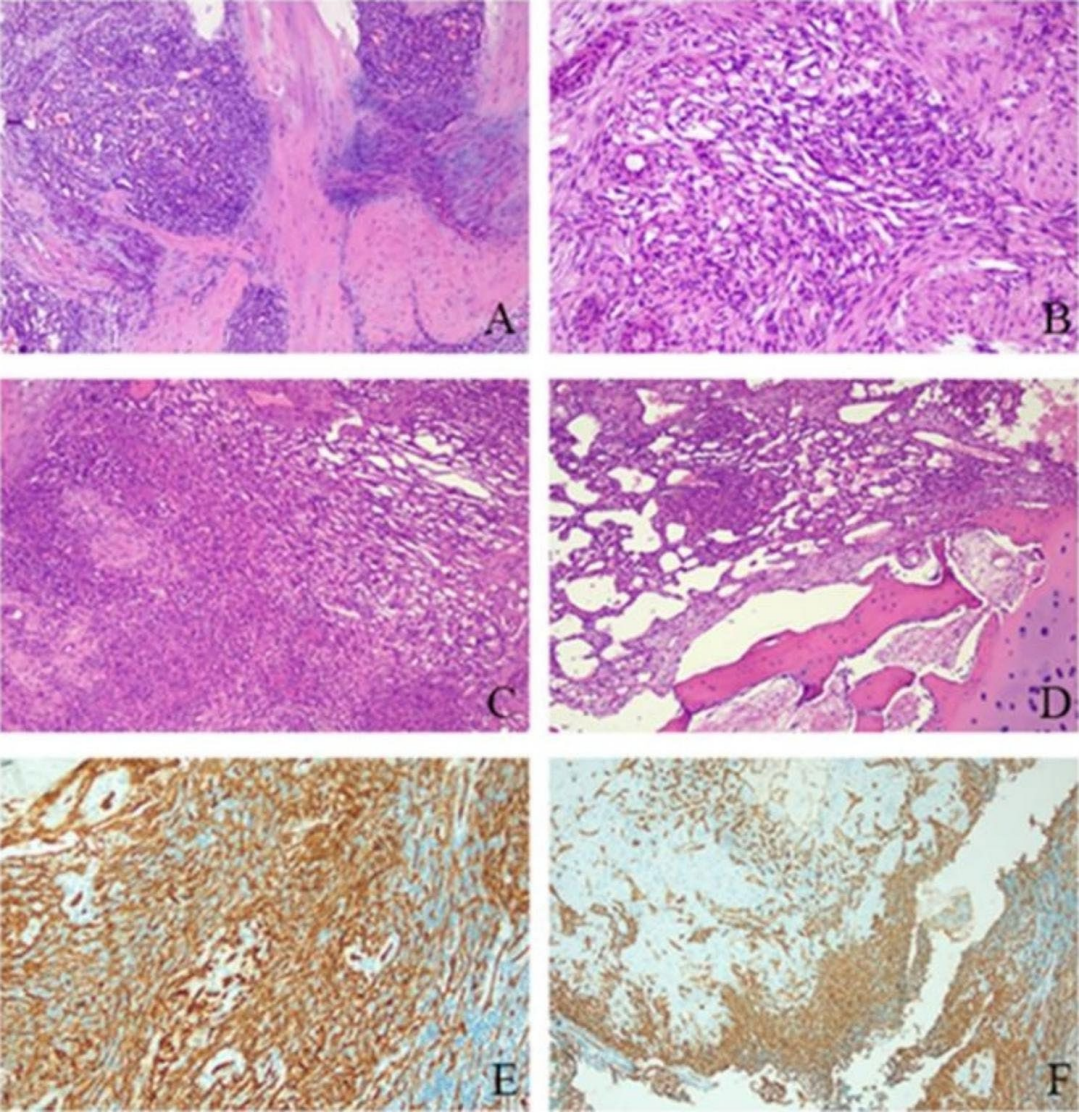
**A**. At low magnification, the area resembled lobulated capillary hemangioma and Kaposiform hemangioendothelioma grow infiltratively in the surrounding soft tissue. **B**. At medium magnification, the area resembled a retiform hemangioendothelioma with spindle-shaped vascular endothelial cells. **C**. At low magnification, the area resembled spindle hemangioendothelioma and epithelioid hemangioendothelioma. **D**. At low magnification, the intramedullary region resembled retiform hemangioendothelioma with bootstrap-like endothelial cells. **E-F**. Immunohistochemical staining showed that the tumor cells were strongly positive for CD31 (**E**) and CD34 (**F**)

A 21-year-old female presented with pain and mild restriction of movement in the root of the left thigh for 2 years, with no significant local swelling, masses, or skin color changes. The patient had a history of muscle strain at the root of the left thigh two years ago. A CT scan revealed a lesion of osteolytic bone destruction in the left pubis with a soft tissue mass (Fig. 1, A). The bone cortex was damaged and reactive osteosclerosis can be seen around the lesion (Fig. 1, B). The lesion showed high signal intensity on the fat-suppressed sequences of T2-weighted MR images and isointense signal intensity on T1-weighted MR images (Fig. 2, A-B), with a size of about 36 mm×35 mm. After injection of the contrast agent (Gd-DTPA), the lesion showed inhomogeneous enhancement (Fig. 2, C). A ^99m^Tc MDP bone scan showed that this lesion was radioactively absent, while the adjacent left acetabulum was radioactively concentrated because of reactive osteosclerosis (Fig. 2, D). ^18^FDG PET-CT showed increased FDG uptake of this lesion with SUVmax of 5.0 (Fig. 2, E-H). A borderline or low-grade malignant tumor was suspected. Finally, the patient underwent radical surgical resection and was pathologically diagnosed with CHE. Microscopically, the tumor was dominated by components of Kaposiform hemangioendothelioma and retiform hemangioendothelioma (Fig. 3, A-B). Areas resembling spindle cell hemangioendothelioma, epithelioid hemangioendothelioma (Fig. 3, C), and retiform hemangioendothelioma with bootstrap-like endothelial cells were also seen (Fig. 3, D). Immunohistochemical results: CD31 (+), CD34 (+), ERG (+), EMA (-), SMA (+), Desmin (-), Ki67 (5% +), CK (-), INI-1 (+), PGMI (-). Among them, CD31(+) and CD34(+) showed extensive and intense positivity (Fig. 3, E-F). No recurrence or metastasis was observed at 5 years postoperative follow-up.

### Case 2

**Fig. 4:**
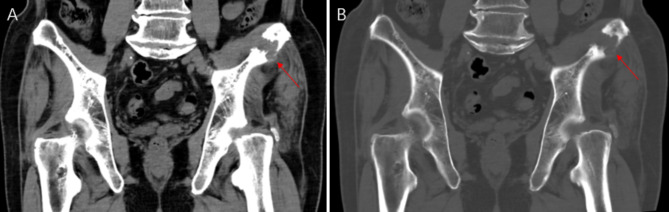
CT revealed a lesion of osteolytic bone destruction in the left ilium with a soft tissue mass (**A**, arrow). The bone cortex was damaged and reactive osteosclerosis can be seen around the lesion (**B**, arrow)

**Fig. 5:**
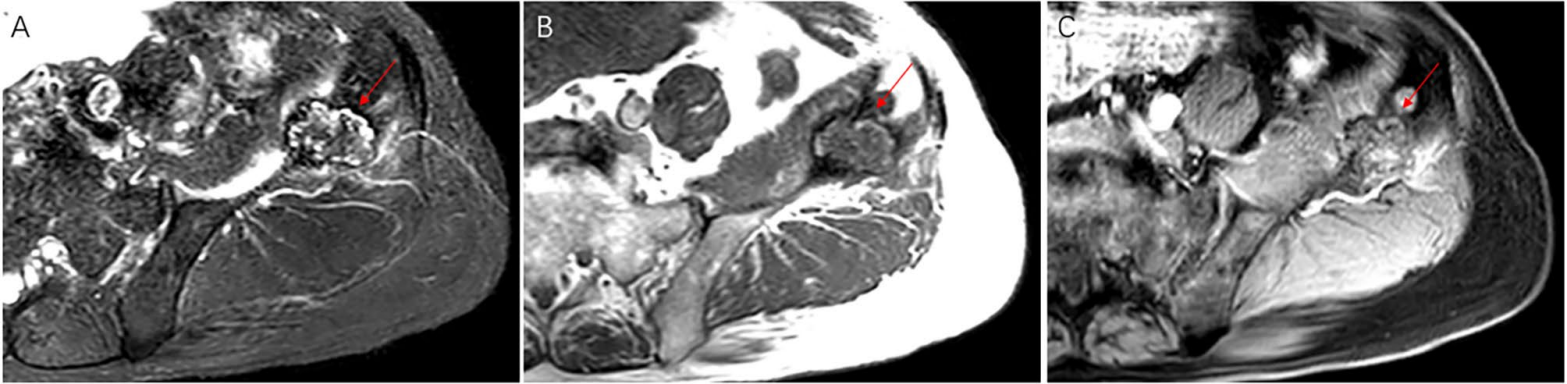
The lesion showed heterogeneous signal on the fat-suppressed sequences of T2-weighted MR images and T1-weighted MR images, with central low signal and peripheral linear high signal (**A-B**, arrow). The size is about 30 mm×26 mm. After injection of the contrast agent (Gd-DTPA), the lesion showed inhomogeneous enhancement (**C**, arrow)

**Fig. 6:**
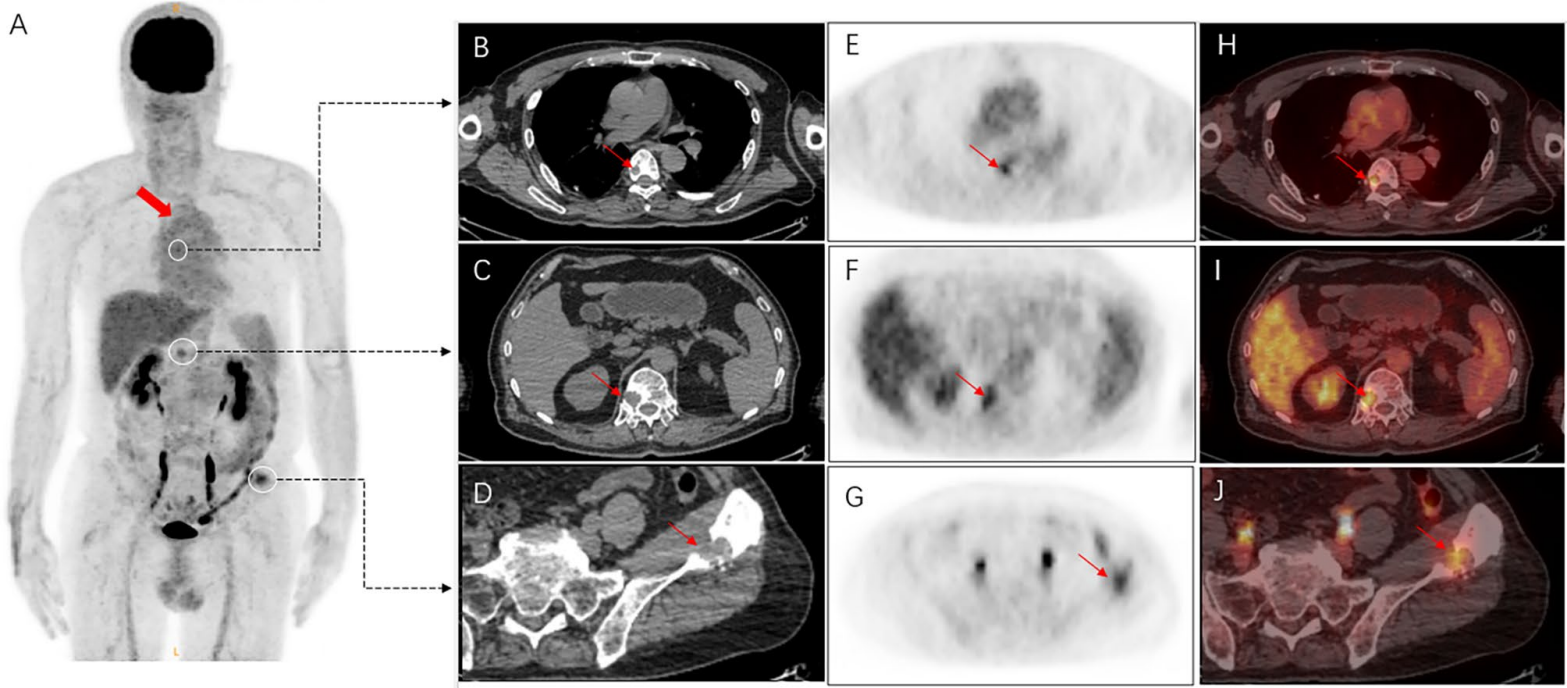
^18^ F-FDG PET/CT identified multiple lesions located in the T6, T12 vertebrae and the left ilium (**A**, white circle), all of which showed osteolytic bone destruction on CT (**B-D**). The maximum density projection (MIP) PET (**A**), corresponding PET (**E-G**), and fusion images (**H-J**) showed increased FDG uptake in these lesions (arrows) with a SUVmax of 4.9. No abnormal FDG uptake was shown on the manubrium sterni (**A**, red arrow)

A 66-year-old male recently presented with pain in the left hip for 6 months, with no significant local swelling, masses, or skin color changes. Ten years ago, the patient underwent surgery for CHE on the manubrium sterni [1]. The present CT scan revealed a lesion of osteolytic bone destruction in the left ilium with a soft tissue mass (Fig. 4, A). The bone cortex was damaged and reactive osteosclerosis can be seen around the lesion (Fig. 4, B). The lesion showed heterogeneous signal on the fat-suppressed sequences of T2-weighted MR images and T1-weighted MR images, with central low signal and peripheral linear high signal(Fig. 5, A-B).The size is about 30 mm×26 mm. After injection of the contrast agent (Gd-DTPA), the lesion showed inhomogeneous enhancement (Fig. 5, C). Apart from the lesion on the left ilium, ^18^ F-FDG PET-CT additionally revealed lesions located on the T6 and T12 vertebrae (Fig. 6, A), all of which showed osteolytic bone destruction on CT (Fig. 6, B-D). FDG uptake was increased in all of these lesions, with a SUVmax of 4.9 (Fig. 6, E-J). No abnormal FDG uptake was shown in the manubrium sterni (Fig. 6, A), and there were no signs of recurrence on CT either.The patient eventually underwent radical surgical resection of the left ilium lesion. The final pathological diagnosis was a CHE. Microscopically, benign cavernous hemangioma, intermediate retiform hemangioendothelioma, and a few highly differentiated angiosarcoma components were seen. Immunohistochemical results: CD31 (+), AE1/AE3 (-), CD34 (+), ERG (+), Ki67 (10% +), TFE-3 (-), CAM5.2 (-); KI67 (1% +). FISH results: FOSB (negative for FOSB-associated gene translocations). It was a pity that the patient did not undergo a pathological biopsy or surgery for the thoracic spine lesions.

## Discussion

Composite hemangioendothelioma (CHE) was first described in 2000 by Nayler et al [2, 5, 7–9]. It is a rare intermediate-grade (occasionally metastatic) vascular tumor that is most often seen in young adults (mean age 42 years) but can present at any age from birth to 75 years, with women being the most common [5, 7–9]. According to reports [10], CHE is commonly associated with underlying vascular abnormalities, including arteriovenous malformations, lymphangioleiomas, and Maffucci’s syndrome. There may be a previous background in radiation therapy or prolonged lymphedema. Comorbid wasting coagulation disorders (Kasabach-Merritt syndrome) and trauma have also been documented [10, 11]. The lesions are mainly located in the distal dermis and subcutaneous soft tissues of the extremities [7, 8, 11–17], and often present as isolated nodules or masses with poorly defined borders [18]. The lesions are variable in color and can be flesh-colored, red, and purple-black [19]. In contrast, the lesions reported here are deeply located with no obvious abnormal changes on the skin surface. In this paper, we report two cases of CHE primitively occurring in the bone, the first case only occurred in the left pubis, while the second case is a patient post-resection of CHE in the manubrium sterni 10 years ago [1], who developed of new lesions in the left ilium and T6, T12 vertebra. The clinical, histopathological, and imaging features of the cases identified so far of bone-derived CHE are compiled in Table 1. CHE has a chronic course with mild early symptoms, mainly presenting as localized dull pain and swelling [4, 19]. All three cases of CHE occurring in bone reported so far occurred in adults and were presented for chronic pain. They were consistent with the presentation of CHE occurring at other sites [1, 2, 5–18, 20–36].

**Table 1:** Clinical, histopathological, and PET-CT features of composite hemangioendothelioma occurring in bone from published cases

Authors	Case	Sex/Age, y	Location	Preoperative duration	CT/MR performance	^18^FDG PET-CT SUVmax	Histological components	Treatment and follow-up
Dong et al.,2014 [1]	1	M,56	Manubrium sterni	2y	MR: high signal intensity on T2-weighted MR images, isointense signal intensity on T1-weighted MR images, with inhomogeneous enhancement.	5.9	EHE, SCH, BH, AS	Surgery, N/A
Perry et al.,2017 [6]	2	F,48	C5 vertebra	N/A	N/A	N/A	N/A	N/A, Met to Lung
Present case	3	F,21	Left Pubis	2y	CT: Osteolytic bone destruction, soft tissue masses, reactive osteosclerosis, damaged bone cortex. MR: high signal intensity on T2-weighted MR images, isointense signal intensity on T1-weighted MR images, with inhomogeneous enhancement.	5.0	CAH, KHE, RHE, SCH, EHE	Surgery, NSR after 5 years
Present case	4*	M,66	Left ilium, T6 and T12 vertebrae(biopsied)	0.5y	CT: Osteolytic bone destruction, soft tissue masses, reactive osteosclerosis, damaged bone cortex. MR: heterogeneous signal on the T2 and T1-weighted MR images, with central low signal and peripheral linear high signal, with inhomogeneous enhancement.	4.9	CH, RHE, AS	The primary lesion in the manubrium sterni underwent surgery 10 years ago; the new lesion in the left ilium also underwent surgery, FIP

CH: cavernous hemangioma; CAH: capillary hemangioma; EHE: epithelioid hemangioendothelioma; KHE: Kaposiform hemangioendothelioma; RHE: retiform hemangioendothelioma; SCH: spindle cell hemangioendothelioma; AS: angiosarcoma; BH: benign hemangioma; N/A: not available, NSR: no sign of recurrence. Met: metastases; *: the same patient as case1, with different disease locations admitted at different hospitals; FIP: follow up in progress

Histopathologically, the CHE components are complicated and includes epithelioid hemangioendothelioma, lymphangioma, cavernous hemangioma, arteriovenous malformation, spindle cell hemangioma, retiform hemangioendothelioma, kaposiform hemangioendothelioma, and others. Besides, low-grade and a small number of angiosarcomas may also be present. Fukunaga et al. [10]reported that the predominant histologic components were histologically identical to epithelioid hemangioendothelioma and retiform hemangioendothelioma. The first case contains both of these typical components. The pathologic components of the left iliac lesion in the second case of CHE includes cavernous hemangioma, retiform hemangioendothelioma, and a small amount of angiosarcoma. These are not identical to the pathological components of the lesion that occurred on manubrium sterni a decade ago. As far as we know, they have only one component in common, angiosarcoma (Table 1). Accordingly, we speculate that the second case is more likely be an asynchronous multifocal growth pattern of CHE [2, 21], but metastasis still can’t be ruled out. Immunohistochemically, the positive expression of the vascular endothelial cell markers CD31 and ERG are relatively specific [37], while the expression of CD34 and lymphatic markers may be variable [6]. The two cases of CHE reported in this paper do not have identical pathological components, but both have the typical component, retiform hemangioendothelioma, and both shows CD31 (+), CD34 (+) ERG (+) on immunohistochemical analysis.

Based on our cases and the cases published so far, there is some commonality in the CT and MR presentation of CHE. Since it is aggressive, it often appears as osteolytic bone destruction with soft tissue masses and cortical destruction of bone on CT. Also, due to its hypervascular nature, it often shows relatively hyperintense T2 signal and can have obvious enhancement after contrast injection. CHE is an intermediate-grade vascular tumor, and the lesion tends to develop slowly, so the margins of the lesion are often visible with varying degrees of sclerosis, which can be differentiated from some malignant tumors. As for the treatment, CT and MRI can also help to determine the tumor margins and assist in determining the extent of preoperative and postoperative radiotherapy [4]. Bone scan seems to lack specificity. In our first case, the lesion on the left pubis presented a loss of radioactivity, while the lesion on the manubrium sterni [1], which occurred previously in the second case, appeared as a hot pot on the whole-body bone scan. Thus, the bone scan has its limitations, and it can lead to misdiagnosis. Therefore MRI/CT is the first imaging method, followed by bone scan can be used as a screening method for multicentricity.

PET-CT is useful in determining the malignancy of tumors that exhibit highly vascular characteristics, but its presentations of CHE has rarely been reported. The lesion on the manubrium sterni that had occurred previously in the second case was reported by Dong et al [1]. ^18^ F-FDG PET/CT showed increased FDG uptake in the lesion with SUVmax of 5.9, which was roughly similar to the lesions we reported here (Table 1). The high FDG uptake of the tumors suggest their malignant potential. In addition, PET-CT examinations allows for evaluation and functional imaging of the systemic situation. This may be important for both staging and follow-up of the disease [1, 5]. Therefore, in clinical practice, we should combine CT, MRI, and PET-CT for a comprehensive evaluation.

There are no standard treatment options for CHE. CHE originating from bone is very rare. There is a lack of research on effective treatment modalities for CHE occurring in bone. Here we list the current effective treatment modalities for CHE in soft tissues in the hope that they may be useful for the treatment of CHE in bone. Complete surgical resection is the conventional treatment for CHE. Other less common therapies, including radiotherapy and chemotherapy, such as electron beam, interferon-α 2b, and thalidomide, are also effective [17, 28]. The local recurrence rate of CHE exceeds 50% [2], therefore, it is recommended to extend the resection appropriately to ensure complete resection of the lesion. Only one case of death during postoperative follow-up has been reported in the English literature so far [6]. Both patients in this article underwent surgeries. The first patient has been followed up for 5 years after surgery without recurrence or metastasis. The second patient who had a surgery for CHE on the manubrium sterni ten years ago [1], is still under follow-up after the second surgery for the left iliac lesion (Table 1). According to the literatures [2, 16], the incidence of lymph node metastasis is about 6% and the incidence of distant metastasis is < 1%. However, two of the three cases of CHE located in the bone had distant metastases or multicentric lesions, indicating the possibility of metastasis or asynchronous growth at multiple sites for CHE originating from bone. This needs to be explored by more extensive research in the future.

## Conclusion

CHE originating from bone is a very rare vascular tumor that is challenging to diagnose preoperatively. Our report provides more detailed information about the diagnostic imaging of CHE. Given that CHE is locally aggressive and occasionally metastatic, PET-CT may be helpful in staging and follow-up. Complete surgical resection is the first choice for treatment.

## Data Availability

All information about the patients came from Shanghai Sixth People’s Hospital Affiliated to Shanghai Jiao Tong University School of Medicine. All data generated or analyzed during this study are included in this published article.

## Abbreviations

CHE: Composite hemangioendothelioma
CT: Computed Tomography
PET-CT: 2-deoxy-2 [F-18] fluoro-D-glucose positron emission tomography-computed tomography
WHO: World Health Organization
